# Reactivity to UV Radiation Exposure Monitoring Using Personal Exposure Devices for Skin Cancer Prevention: Longitudinal Observational Study

**DOI:** 10.2196/29694

**Published:** 2021-09-28

**Authors:** Bridget G Parsons, Elizabeth S Nagelhout, Ali P Wankier, Nan Hu, Riley Lensink, Angela Zhu, Katy Nottingham, Douglas Grossman, Jakob D Jensen, Yelena P Wu

**Affiliations:** 1 Huntsman Cancer Institute University of Utah Salt Lake City, UT United States; 2 Department of Public Health Westminster College Salt Lake City, UT United States; 3 Department of Biostatistics Robert Stempel College of Public Health & Social Work Florida International University Miami, FL United States; 4 Department of Communication University of Utah Salt Lake City, UT United States; 5 Department of Dermatology University of Utah Salt Lake City, UT United States

**Keywords:** ultraviolet radiation exposure, wearable device, melanoma, melanoma prevention, mHealth, digital health, eHealth, UVR monitoring, mobile phone

## Abstract

**Background:**

Emerging UV radiation (UVR) monitoring devices may present an opportunity to integrate such technology into skin cancer prevention interventions. However, little is known about the effects of using a wearable UVR monitor on adults’ and children’s sun protection–related behaviors and attitudes (eg, cancer worry and perceived risk). Understanding the potential role of reactivity and seasonal effects will help inform the use of objective monitors in the context of skin cancer prevention research, including intervention studies.

**Objective:**

The aim of this study is to examine the potential reactivity associated with a wearable personal UVR monitor, specifically the effects associated with reported sun-protective behaviors and skin cancer–related attitudes, which are often the targets of skin cancer preventive interventions.

**Methods:**

Child-parent dyads (n=97 dyads) were asked to wear a UVR monitoring device during waking hours for 2 weeks. Participants were asked to sync the device daily with a smartphone app that stored the UVR exposure data. Participants were blinded to their UVR exposure data during the 2-week period; thus, the smartphone app provided no feedback to the participants on their UVR exposure. Participants completed self-report questionnaires assessing sun-protective behaviors, sunburn, tanning, skin self-examination, skin cancer–related knowledge, perceived risk, cancer worry, response efficacy, and intentions to change behaviors over the 2-week period. Linear regressions were conducted to investigate changes in the outcomes over time and to account for the role of the season of study participation.

**Results:**

Regression results revealed that there was a significant decrease over time for several sun protection outcomes in children, including time spent outdoors on weekends (*P*=.02) and weekdays (*P*=.008), sunscreen use (*P*=.03), reapplication (*P*<.001), and unintentional tanning (*P*<.001). There were no significant changes over time in children’s and parents’ UVR exposure, sunburn occurrence, or sun protection attitudes. Season of participation was associated with several outcomes, including lower sunscreen use (*P*<.001), reapplication (*P*<.001), sunburns (*P*=.01), intentions to change sun-protective behaviors (*P*=.02), and intentional (*P*=.008) and unintentional tanning (*P*=.01) for participants who participated in the fall versus the summer.

**Conclusions:**

The findings from this study suggest that daily use of a UVR monitoring device over a 2-week period may result in changes in certain sun-protective behaviors. These results highlight the importance of identifying and addressing potential reactivity to UVR monitoring devices, especially in the context of skin cancer preventive intervention research. Ultimately, objectively assessed UVR exposure could be integrated into the outcome assessment for future testing of skin cancer prevention interventions.

## Introduction

### Background

Mobile or personal health monitors are frequently used by the general public and in the context of health behavior research [1]. For example, FitBit, one of the most popular wearable activity monitors, reported 2 million new users in 2019, bringing its total active users to 29.6 million [2]. In the past decade, >300 studies using FitBit as an outcome measure were registered with the National Institutes of Health [3]. Although physical activity monitors are frequently used in research settings, monitors related to environmental exposures and their associated health behaviors are in the early phases of development, testing, and dissemination [4-6]. In the context of skin cancer prevention, assessment of an individual’s UV radiation (UVR) exposure is essential and is becoming more feasible given the availability of personal UVR monitors.

Melanoma is the deadliest type of skin cancer and the fifth most common form of cancer [7]. UVR exposure from the sun or artificial sources is the primary modifiable risk factor for melanoma [8]. Efforts to prevent melanoma focus on decreasing individuals’ UVR exposure through sun-protective behaviors, such as wearing sunscreen and clothing that covers the skin, and avoiding peak UVR hours, which are typically assessed through self-report measures [9]. There has been a growing number of objective UVR assessment methods that can quantify personal UVR exposure and be useful for documenting the efficacy of melanoma preventive interventions [4,10]. However, a potential concern with using objective UVR exposure assessments is reactivity or the possibility that simply monitoring a behavior may in and of itself result in behavior change [11,12]. For example, it is possible that individuals who are aware that their UVR exposure is being monitored could choose to use sun-protection methods more consistently. For example, in the physical activity assessment literature, it has been found that after individuals use objective assessments of their exercise behaviors, changes can be seen in both their behaviors and exercise-related attitudes (eg, covering more distance and reporting great perceived exertion) [13]. In addition, it is not known to what extent personal UVR exposure based on objective assessments could differ depending on environmental factors such as the season. For instance, individuals may make different sun-protection choices based on seasonal differences in weather and temperature [14]. Studies have found that individuals who rely on ambient temperature as a method for determining the need for sun protection, particularly in the winter [15,16], are more likely to receive sunburns in cool weather [17] and tend to use clothing such as long sleeves and pants for warmth rather than sun protection [14].

Although prior studies of objective UVR exposure monitors have not yet examined potential reactivity effects, this phenomenon has been more fully explored in physical activity research [18]. In the literature, there are mixed findings regarding whether monitoring behavior alone is associated with behavior change. Most studies reporting on behavioral changes among adults and children related to monitoring used devices such as pedometers that provided feedback to the participants on physical activity outcomes (eg, number of steps taken daily) [19-22]. In the studies of monitors that did not provide feedback, most found that monitoring alone was not associated with behavior change [23-26].

### Objective

In relation to melanoma prevention, studies have examined the effects of providing periodic feedback to adults, adolescents, and children on UVR exposure; information on the risk of sunburn; and advice about sun protection methods [27-29]. For example, the provision of UVR exposure feedback is associated with less UVR exposure on weekends and an increase in the use of some forms of sun protection [27-29]. Other studies have examined variability in UVR exposure and sun-protective behaviors among different populations, such as rural and urban children, and periods, such as during vacation [30-34]. However, it remains unknown to what extent UVR measurement itself, in the absence of providing feedback on UVR exposure, is associated with changes in sun-protective behaviors and attitudes (eg, cancer worry and perceived risk) in adults and children. Some sun-protection behaviors (eg, sunscreen use, hat use, and wearing pants) are not directly measured by the device. Our hypothesis, based on the literature on physical activity and limited UVR exposure reactivity, is that wearing a UVR monitoring device could lead individuals to become more aware of their UVR exposure and, as a result, change their behaviors and attitudes related to sun exposure. For example, in the physical activity literature, attitudes related to exercise such as perceived efficiency, intensity of effort, and fatigue have been found to increase with accelerometer use [13]. We hypothesize that sun-protection behaviors increase over the monitoring period and that UVR measured by the device would decrease as a result of increased UVR exposure awareness. Understanding the potential role of reactivity and seasonal effects will help inform the use of objective monitors in the context of skin cancer prevention research, including intervention studies. This pilot study was designed to validate the use of a UVR monitoring device in larger skin cancer prevention interventions designed for parents and children [5]. The goal of this study is to assess the potential reactivity associated with UVR exposure monitoring among adults and children as it relates to sun-protective behaviors and skin cancer prevention–related attitudes, which are often the targets of skin cancer preventive interventions [35-37].

## Methods

### Participants

Potential participants were recruited through health and community events (eg, health fairs and farmers’ markets) and invitation letters that were mailed to residents with a child aged between of 8 and 17 years. A web-based marketing resource was used to obtain the addresses of potential participants living in Utah [38]. To be eligible to participate in this study, adults who were residents of Utah, aged ≥18 years, had at least one child aged between 8 and 17 years, had a smartphone with Bluetooth and Wi-Fi capabilities, were willing to use a smartphone app that shared their UVR exposure information with the research team, did not have a pacemaker (because of potential interference from the UVR monitor), and were able to read and write in English were included. For children to be eligible to participate, they had to be aged between 8 and 17 years, live with a primary caregiver in Utah, have no previous melanoma diagnosis, and not have a pacemaker. In total, 224 adults expressed interest in participating in the study. Of the 224 adults, 150 (67.0%) were screened for eligibility. Of the 150 adults screened, 116 (77.3%) were screened as eligible and 34 (22.7%) as ineligible. Reasons for ineligibility included not having a child in the desired age range (28/34, 82%), not having a smartphone with the necessary specifications (5/34, 15%), and not being able to read or write in English (1/34, 3%). A total of 97 parents (77% biological mothers) and 97 children (mean age 12.7 years, SD 2.7) were enrolled between June 2018 and October 2018. These months were selected because of the high ambient UVR levels in the region during those months [39]. All study procedures were approved by the relevant institutional review board.

### Procedures

Participants were asked to wear a UVR monitoring device (Shade wearable UVR sensor, model V1.00, YouV Labs Inc) [4,40] on their clothing for a 2-week period. They completed the baseline assessment before being given the UVR monitoring devices to wear. A monitoring period of 2 weeks was selected to allow the capture of data from both weekdays and weekends for >1 week (to minimize missing data for either weekdays or weekend days). Participants were asked to synchronize the Shade device daily with a smartphone app that stored the UVR exposure data in a cloud-based server accessed by the research team. Participants were blinded to their UVR exposure data during the 2-week period; thus, the smartphone app provided no feedback to the participants on their UVR exposure. Changes in UVR exposure were analyzed by comparing total UVR exposure in the first week of study participation with total UVR exposure in the second week of study participation. Participants were asked to complete electronically delivered questionnaires via REDCap (Research Electronic Data Capture) [41] at the beginning and end of the 2-week period about sun-protective behaviors—such as wearing sunscreen and protective clothing (10 items) [42], number of sunburns received (one item) [42], tanning (two items) [43], skin self-examination (one item) [44], skin cancer–related knowledge (10 items) [45]—and relevant attitudes such as cancer worry (four items) [46], perceived risk (two items) [47], efficacy of skin cancer preventive behaviors (four items) [48], and intentions to change preventive behaviors (five items) [49-51]. Parents reported on these constructs for both themselves and their child, and children provided self-reports. All items except knowledge (true or false items) were asked on a 5-point Likert scale. The multi-item scales, including skin cancer–related knowledge, cancer worry (α=.87, child report; α=.90, parent report; parents not asked to report on child), perceived risk (α=.75, child report; α=.82, parent report; α=.84, parent report on child), efficacy of preventive behaviors (α=.89, child report; α=.94, parent report; α=.95, parent report on child), and intention to change preventive behaviors (α=.39, child report; α=.55, parent report; α=.66, parent report on child), were summed. After completing their study participation, each participant was provided with a gift card and a report summarizing their UVR exposure over the 2-week period.

### Analyses

Descriptive statistics were used to summarize the participants’ demographic information and summary statistics for the outcomes of interest. Linear regressions were used to examine potential changes over the 2-week period in UVR exposure measured from the device, sun-protective behaviors, sunburn, tanning, skin self-examination, and in summed scales for skin cancer–related knowledge, perceived risk, cancer worry, response efficacy, and intentions to change behaviors. Additional linear regressions were conducted with seasonal (summer [June-August] vs fall [September-October]) assessment time points (baseline vs exit) and their interactions as predictors and the same outcomes as dependent variables [52]. Season was included in these models based on the existing literature [53], and it was significantly related to multiple outcomes in this study.

## Results

### Sample Characteristics

In total, 97 parent-child dyads participated in the study. Of the 97 parents, 73 (77%) were biological mothers, 83 (87%) were non-Hispanic White, and 5 (5%) were Hispanic. Of the 97 children (mean age 12.7 years, SD 2.7), 81 (85%) were non-Hispanic White, 8 (8%) were Hispanic, and 56 (59%) were female (Table 1).

**Table 1:** Demographic characteristics of participating parents and children (N=97)^a^.

Characteristics			Parents		Children
Age (years), mean (SD)			41.6 (6.3)		12.7 (2.7)
Gender (female), n (%)			73 (77)		56 (59)
**Race, n (%)**					
	Non-Hispanic White	83 (87)		81 (85)	
	Hispanic	5 (5)		8 (8)	
	Asian or Asian American	5 (5)		4 (4)	
	Other	2 (2)		2 (2)	
**Fitzpatrick skin type (I-VI)**					
	Type I	9 (9)		2 (2)	
	Type II	15 (15)		17 (17)	
	Type III	42 (43)		41 (42)	
	Type IV	24 (25)		32 (33)	
	Type V	5 (5)		3 (3)	
**Marital status, n (%)**					
	Married or marriage-like relationship	84 (88)		N/A^b^	
	Divorced/separated	9 (10)		N/A	
	Widowed	2 (2)		N/A	
**Level of education, n (%)**					
	High school graduate or GED^c^	8 (8)		N/A	
	Some college, including 2-year degree	41 (42)		N/A	
	Bachelor’s degree	25 (26)		N/A	
	Master’s/doctoral degree	21 (22)		N/A	
**Household income (US** **$** **), n (%)**					
	<50,000	23 (24)		N/A	
	>50,000	64 (67)		N/A	
	Would prefer not to report	8 (8)		N/A	

^a^Two families did not complete the baseline questionnaire, thus each of the categories have a total of 95.

^b^N/A: not applicable (children were not asked this question).

^c^GED: General Education Development.

### Descriptive Statistics

Descriptive statistics (means and SDs) for UVR exposure, sun-protective and risk behaviors, and skin cancer–related attitudes are presented in Table 2.

**Table 2:** Descriptive statistics for UV radiation exposure and sun-protective behaviors, knowledge, and attitudes.

	Child self-report			Parent report on child			Parent self-report	
	Baseline, mean (SD)	Exit, mean (SD)	Baseline, mean (SD)		Exit, mean (SD)	Baseline, mean (SD)		Exit, mean (SD)
UVR^a^ exposure^b^	6.00 (6.09)	4.76 (5.27)	N/A^c^		N/A	6.89 (6.38)		6.11 (6.62)
UVR exposure (adjusted)^d^	6.47 (5.94)	5.50 (5.07)	N/A		N/A	7.56 (6.58)		6.87 (6.81)
Hours outdoors: weekday	2.69 (1.75)	2.24 (1.65)	2.72 (1.47)		2.09 (1.43)	2.79 (1.80)		2.01 (1.68)
Hours outdoors: weekend	3.22 (2.09)	2.49 (1.57)	3.55 (1.97)		2.76 (1.68)	3.88 (1.91)		2.72 (1.66)
Sunscreen^e^	2.35 (1.45)	1.83 (1.13)	2.52 (1.39)		2.02 (1.21)	2.53 (1.45)		2.11 (1.33)
Reapplication^e^	1.95 (1.15)	1.50 (0.84)	1.98 (1.19)		1.60 (0.86)	1.87 (1.19)		1.60 (0.96)
Long sleeves^e^	1.76 (0.93)	2.12 (1.03)	1.86 (1.00)		2.16 (1.04)	1.87 (1.06)		2.20 (1.15)
Pants^e^	2.76 (1.21)	3.03 (1.36)	2.52 (1.19)		2.82 (1.32)	3.02 (1.34)		3.19 (1.37)
Hat^e^	1.31 (0.82)	1.37 (0.91)	1.37 (0.73)		1.37 (0.90)	1.97 (1.33)		1.84 (1.07)
Shade^e^	2.56 (1.04)	2.51 (1.06)	2.45 (0.96)		2.43 (1.01)	2.67 (1.00)		2.55 (1.02)
Avoid peak hours^e^	2.08 (0.94)	2.14 (1.02)	2.33 (1.19)		2.34 (1.04)	2.55 (1.14)		2.35 (1.00)
Sunglasses^e^	1.92 (1.18)	1.59 (1.01)	1.89 (1.18)		1.60 (0.99)	3.58 (1.40)		3.19 (1.34)
Sunburn	0.23 (0.57)	0.16 (0.43)	0.15 (0.36)		0.16 (0.42)	0.10 (0.30)		0.18 (0.44)
Intentional tanning^e^	1.24 (0.63)	1.15 (0.47)	1.16 (0.45)		1.09 (0.33)	1.13 (0.48)		1.08 (0.31)
Unintentional tanning^e^	2.88 (1.17)	1.80 (1.07)	2.95 (1.33)		1.80 (1.02)	2.93 (1.16)		1.90 (1.05)
Indoor tanning^e^	1.00 (0)	1.00 (0)	1.00 (0)		1.00 (0)	1.00 (0)		1.00 (0)
Skin self-examination^e^	1.88 (0.32)	1.88 (0.32)	0.09 (0.29)		0.17 (0.38)	0.16 (0.37)		0.12 (0.33)
Skin cancer knowledge^f^	2.98 (1.42)	3.23 (1.49)	N/A		N/A	4.08 (1.01)		4.24 (0.95)
UVR knowledge^f^	2.97 (1.27)	3.31 (1.13)	N/A		N/A	3.84 (0.67)		3.88 (0.65)
Total knowledge^f^	5.94 (2.35)	6.55 (2.25)	N/A		N/A	7.92 (1.29)		8.11 (1.22)
Perceived risk^g^	4.52 (1.68)	4.48 (1.79)	5.67 (1.75)		5.83 (1.60)	6.33 (1.88)		6.19 (1.83)
Cancer worry^h^	7.36 (3.34)	7.10 (3.20)	N/A		N/A	9.52 (3.73)		9.12 (3.52)
Response efficacy^i^	15.29 (3.44)	15.40 (3.81)	16.53 (3.84)		17.3 (2.74)	17.00 (3.57)		17.37 (2.89)
Intentions to change^j^	10.66 (3.93)	10.92 (4.53)	11.35 (4.73)		11.74 (4.89)	13.66 (5.04)		13.38 (4.96)

^a^UVR: UV radiation.

^b^First 7 days versus last 7 days of the 2-week wearing period.

^c^N/A: not applicable (parents not asked to report this on children).

^d^First 7 days versus last 7 days of the 2-week wearing period adjusted to include participants that had at least 4 days of UVR data.

^e^Response options included never=1 to always=5.

^f^0=false, 1=true. Possible range of 0-5, or 0-10 for total knowledge.

^g^1=very unlikely, 5=very likely. Possible range of 1-10.

^h^1=not at all, 5=very much. Possible range of 1-20.

^i^1=strongly disagree, 5=strongly agree. Possible range of 1-20.

^j^1=no and I do not intend to start doing so in the next 6 months, 5=yes, I have been for more than 6 months. Possible range of 1-25.

### Regression Analyses

Regression results revealed that there was a significant change over time for several outcomes (Tables 3-5). In addition, separate analyses examining the relationship between season alone and these outcomes are provided in the table footnotes.

**Table 3:** Child self-report regression models examining association between time and season of participation and sun-protective behaviors^a^.

Outcome	*R* ^2^	*F* test (*df*)	Time						Season				
			ß	SE (ß)	B	*t* test	*P* value	ß		SE (ß)	B	*t* test	*P* value
UVR^b^ exposure^c^	0.00	0.17 (181)	0	1.17	0.00	0	.99	–.05		1.29	–0.64	–0.49	.62
UVR exposure adjusted^d^	0.01	0.38 (137)	–.06	1.26	–0.70	–0.56	.58	–.04		1.30	–0.42	–0.33	.75
Hours outdoors: weekday	0.02	1.36 (181)	–.17	0.33	–0.59	–1.77	.08	–.11		0.36	–0.39	–1.06	.29
Hours outdoors: weekend	0.04	2.59 (181)	–.16	0.37	–0.61	–1.67	.09	.01		0.39	0.02	0.04	.96
Sunscreen	0.29	23.62 (181)	–.22	0.22	–0.59	–2.69	.008	–.56		0.24	–1.52	–6.39	<.001
Reapplication	0.18	13.10 (181)	–.28	0.18	–0.57	–3.12	.002	–.43		0.20	–0.92	–4.60	<.001
Long sleeves	0.06	3.83 (181)	.07	0.19	0.14	0.76	.45	–.01		0.20	–0.01	–0.06	.95
Pants	0.05	3.29 (181)	.08	0.24	0.19	0.81	.42	.18		0.26	0.45	1.74	.08
Hat	0.02	1.48 (181)	.01	0.17	0.02	0.11	.92	–.15		0.18	-0.27	-1.46	.15
Shade	0.05	3.22 (181)	.08	0.20	0.17	0.88	.34	–.11		0.22	-0.23	-1.09	.28
Avoid peak hours	0.07	4.63 (181)	.17	0.18	0.34	1.89	.06	–.02		0.20	–0.03	–0.16	.87
Sunglasses	0.06	3.64 (181)	–.14	0.21	–0.32	–1.52	.13	–.14		0.23	–0.32	–1.41	.16
Sunburn	0.09	5.64 (181)	–.12	0.09	–0.12	–1.29	.20	–.36		0.10	–0.36	-3.65	<.001
Intentional tanning	0.04	2.43 (181)	–.09	0.11	–0.10	–0.96	.34	–.18		0.11	–0.20	–1.75	.08
Unintentional tanning	0.18	13.42 (181)	–.43	0.22	–1.06	–4.88	<.001	–.12		0.24	–0.27	–1.15	.25
Indoor tanning	—^e^	—	—	—	—	—	—	—		—	—	—	—
Skin self-exam	0.01	0.70 (181)	.05	0.06	0.03	0.50	.62	.13		0.07	0.09	1.26	.21
Skin cancer knowledge	0.01	0.68 (181)	.03	0.28	0.09	0.33	.75	.01		0.31	0.03	0.09	.93
UVR knowledge	0.02	1.21 (181)	.12	0.24	0.30	1.25	.39	.09		0.26	0.22	0.85	.39
Total knowledge	0.02	1.13 (181)	.08	0.45	0.39	0.87	.39	.05		0.49	0.25	0.51	.61
Perceived risk	0.01	0.07 (181)	–.03	0.34	–0.03	–0.11	.92	.01		0.37	0.01	0.01	.99
Cancer worry	0.01	0.79 (181)	–.08	0.63	–0.52	–0.82	.41	–.11		0.69	–0.76	–1.09	.27
Response efficacy	0.01	0.35 (181)	.01	0.69	0.01	0.01	.99	–.07		0.75	–0.54	–0.72	.48
Intentions to change	0.09	5.50 (181)	.06	0.78	0.46	0.59	.56	–.22		0.85	–1.89	–2.23	.03

^a^Models examining the relationship between season alone and the outcomes of interest indicated that shade seeking, avoidance of peak hours, wearing sunglasses, and intentional tanning were lower in the fall versus summer, whereas pants and hat wearing were higher in the fall.

^b^UVR: UV radiation.

^c^First 7 days versus last 7 days of the 2-week wearing period.

^d^First 7 days versus last 7 days of the 2-week wearing period adjusted to include participants who had at least 4 days of UVR data.

^e^No indoor tanning was reported.

**Table 4:** Parent report on child regression models examining the association between time and season of participation and sun-protective behaviors^a^.

Outcome	*R* ^2^	*F* test (df)	Time						Season				
			ß	SE (ß)	B	*t* test	*P* value	ß		SE (ß)	B	*t* test	*P* value
Hours outdoors: weekday	0.08	5.16 (183)	–.22	0.28	–0.68	–2.42	.02	–.16		0.31	–0.48	1.56	.12
Hours outdoors: weekend	0.07	4.30 (183)	–.25	0.35	–0.94	–2.68	.008	–.14		0.38	–0.55	–1.44	.15
Sunscreen	0.23	18.26 (183)	–.18	0.23	–0.51	2.17	.03	–.41		0.26	–1.16	–4.53	<.001
Reapplication	0.20	14.86 (183)	–.29	0.21	–0.69	–3.31	<.001	–.47		0.23	–1.15	–5.02	<.001
Long sleeves	0.04	2.29 (183)	–.02	0.21	0.03	0.16	.87	–.10		0.23	–0.24	–1.02	.31
Pants	0.04	2.47 (183)	–.01	0.24	–0.03	–0.11	.91	–.05		0.28	–0.13	–0.49	.62
Hat	0.01	0.23 (183)	–.02	0.17	0.04	–0.24	.81	.03		0.19	–0.06	–0.33	.74
Shade	0.08	5.19 (183)	.04	0.20	0.09	0.45	.66	–.19		0.22	–0.41	–1.86	.07
Avoid peak hours	0.03	1.97 (183)	.01	0.24	0.03	0.11	.91	–.12		0.26	–0.31	1.17	.25
Sunglasses	0.02	1.33 (183)	–.14	0.24	–0.33	–1.42	.16	–.07		0.26	–0.18	–0.68	.50
Sunburn	0.06	3.57 (183)	–.01	–0.07	–0.01	–0.02	.98	–.26		0.08	–0.20	–2.53	.01
Intentional tanning	0.05	3.35 (183)	–.17	0.09	–0.18	–1.86	.07	–.27		0.11	–0.29	–2.67	.008
Unintentional tanning	0.24	18.58 (182)	–.48	0.23	–1.30	–5.72	<.001	–.24		0.25	0.66	–2.62	.01
Indoor tanning	—^b^	—	—	—	—	—	—	—		—	—	—	—
Skin self-exam	0.01	0.75 (183)	–.12	0.07	–0.08	–1.27	.21	–.03		0.07	–0.02	–0.25	.81
Perceived risk	0.03	2.04 (183)	.10	0.32	0.33	1.04	.30	.23		0.35	0.76	2.20	.03
Response efficacy	0.02	0.94 (183)	.16	0.64	1.05	1.65	.10	.07		0.70	0.48	0.69	.49
Intentions to change	0.07	4.42 (183)	.05	0.89	0.43	0.48	.63	–.24		0.97	–2.35	–2.42	.02

^a^Models examining the relationship between season alone and the outcomes of interest indicated that shade seeking, avoidance of peak hours, and hours spent outside on weekdays were lower in the fall versus summer, whereas pants wearing was higher in the fall.

^b^No indoor tanning was reported.

**Table 5:** Parent self-report regression models examining the association between time and season of participation and sun-protective behaviors^a^.

Outcome	*R* ^2^	*F* test (*df*)	Time						Season				
			ß	SE (ß)	B	*t* test	*P* value	ß		SE (ß)	B	*t* test	*P* value
UVR^b^ exposure^c^	0.02	0.92 (179)	–.08	1.24	–1.05	–0.85	.40	–.14		1.38	–1.85	–1.34	.18
UVR exposure adjusted^d^	0.01	0.64 (162)	–.08	1.30	–1.05	–0.81	.42	–.14		1.45	–1.87	–1.29	.20
Hours outdoors: weekday	0.01	0.32 (177)	.01	0.33	0.01	0.01	.99	–.07		0.36	–0.25	–0.70	.49
Hours outdoors: weekend	0.02	0.98 (183)	.04	0.33	0.14	0.42	.67	–.06		0.36	–0.19	–0.54	.59
Sunscreen	0.16	11.44 (183)	–.16	0.25	–0.44	–1.79	.08	–.40		0.27	–1.13	–4.21	<.001
Reapplication	0.14	9.91 (183)	–.14	0.19	–0.31	–1.55	.12	–.36		0.22	–0.82	–3.78	<.001
Long sleeves	0.04	2.28 (183)	.04	0.21	0.09	0.43	.67	–.09		0.23	–0.20	–0.87	.39
Pants	0.06	3.32 (183)	–.02	0.25	–0.05	–0.18	.86	.10		0.28	0.26	0.95	.35
Hat	0.01	0.41 (183)	–.03	0.23	–0.08	–0.33	.74	–.05		0.25	–0.13	–0.52	.60
Shade	0.08	5.43 (183)	.01	0.18	0.02	0.13	.89	–.16		0.20	–0.33	–1.61	.11
Avoid peak hours	0.05	2.99 (183)	.04	0.20	0.08	0.39	.70	–.01		0.22	–0.03	–0.12	.91
Sunglasses	0.02	1.37 (183)	–.16	0.26	–0.44	–1.68	.09	–.03		0.29	–0.08	–0.27	.79
Sunburn	0.03	2.03 (183)	.16	0.07	0.12	1.71	.09	–.06		0.08	–0.04	–0.56	.58
Intentional tanning	0.01	0.58 (183)	–.08	0.08	–0.06	–0.83	.41	–.09		0.08	–0.08	–0.94	.35
Unintentional tanning	0.22	16.52 (183)	–.37	0.20	–0.88	–4.33	<.001	–.14		0.22	–0.35	–1.57	.12
Indoor tanning	—^e^	—	—	—	—	—	—	—		—	—	—	—
Skin self-exam	0.01	0.88 (183)	.04	0.07	0.03	0.45	.65	.03		0.07	0.02	0.24	.81
Skin cancer knowledge	0.01	0.26 (183)	.04	0.19	0.09	0.46	.65	–.03		0.21	–0.06	–0.29	.77
UVR knowledge	0.01	0.09 (183)	.01	0.13	0.01	0.08	.94	–.05		0.14	–0.06	–0.44	.66
Total knowledge	0.01	0.27 (183)	.04	0.24	0.09	0.40	.69	–.05		0.26	–0.12	–0.47	.64
Perceived risk	0.01	0.84 (183)	–.03	0.35	–0.12	–0.34	.73	.12		0.39	0.45	1.15	.25
Cancer worry	0.02	0.91 (183)	–.05	0.70	–0.36	–0.52	.60	.12		0.77	0.87	1.13	.26
Response efficacy	0.01	0.33 (183)	.06	0.62	0.41	0.66	.51	–.03		0.68	–0.20	–0.30	.77
Intentions to change	0.02	2.01 (183)	.03	0.95	0.26	0.27	.79	–.07		1.05	–0.68	–0.65	.52

^a^Models examining the relationship between season alone and the outcomes of interest indicated that shade seeking, unintentional, and intentional tanning were lower in the fall versus summer.

^b^UVR: UV radiation.

^c^First 7 days versus last 7 days of the two-week wearing period.

^d^First 7 days versus last 7 days of the two-week wearing period adjusted to include participants that had at least 4 days of UVR data.

^e^No indoor tanning was reported.

There was a significant decrease in children’s sunscreen use based on child (*F*_3,178_=23.62; *P*<.001; *R*^2^=0.29) and parent report (*F*_3,180_=18.21; *P*<.001; *R*^2^=0.23). When season was held constant, sunscreen use decreased in children over the 2-week study period based on child (ß*=*–.22; *t*_3_=–2.69; *P*=.008) and parent report (ß=–.18; *t*_3_=–2.17; *P*=.03). There were also decreases in reapplication of sunscreen in children based on child (ß=–.28; *t*_3_=–3.12; *P*=.002) and parent report (ß=–.69; *t*_3_=–3.31; *P*<.001). There was a significant decrease in reported unintentional tanning for children based on child report (ß=–.43; *t*_3_=–4.88; *P*<.001) and parent report (ß=–.48; *t*_3_=–5.72; *P*<.001) and parents (ß=–.37; *t*_3_=–4.33; *P*<.001). In addition, there were decreases in the hours children spent outside on weekdays (ß=–.22; *t*_3_=–2.42; *P*=.02) and weekends (ß=–.25; *t*_3_=–2.68; *P*=.008) based on parent report. There were no significant changes over time in UVR exposure, sunburn, or attitudes.

Season of participation was associated with several outcomes. Reported sunscreen use was lower for children based on child (ß=–.56; *t*_3_=–6.39; *P*<.001) and parent report (ß=–.41; *t*_3_=–4.53; *P*<.001) and parents (ß=–.40; *t*_3_=–4.21; *P*<.001) who participated in fall versus summer*.* Reapplication of sunscreen was also lower for children based on child (ß=–.43; *t*_3_=–4.60; *P*<.001) and parent report (ß=–.47; *t*_3_=–5.02; *P*<.001) and parents (ß=–.36; *t*_3_=–3.78; *P*<.001) who participated in fall. Reported child sunburns per child (ß=–.36; *t*_3_=–3.65; *P*<.001) and parent report (ß=–.26; *t*_3_=–2.53; *P*=.01) and intentions to change were lower for children who participated in the fall (ß=–.22; *t*_3_=–2.23; *P*=.03) than in the summer (ß=–.24; *t*_3_=–2.42; *P*=.02). Intentional tanning (ß=–.27; *t*_3_=–2.67; *P*=.008) and unintentional tanning (ß=–.24; *t*_3_=–2.62; *P*=.01) were lower for children who participated in the fall based on parent report*.* Perceived risk for cancer was higher for children who participated in the fall compared with those who participated in summer based on parent report (ß=.23; *t*_3_=2.20; *P*=.03).

## Discussion

### Principal Findings

The current findings indicate that it may be important to identify and address the reactivity to UVR monitoring devices among children and parents. Our results provide initial evidence that the use of a UVR monitoring device may be associated with changes in sun-protective behaviors. In the context of intervention studies seeking to improve the use of sun-protection behaviors, the reactivity effects observed, particularly the decreased use of sun-protection behaviors, could potentially dampen the detection of desired intervention effects.

In contrast to most previous studies that found that monitoring alone did not lead to behavior change, some significant changes in skin cancer preventive behaviors over time were detected, including in children’s time spent outside on weekdays and weekends, use and reapplication of sunscreen, and unintentional tanning. Notably, almost all of the changes in outcomes observed were decreases in reported sun-protective behaviors over time. This could indicate that wearing the UVR monitoring device initially led participants to increase their use of sun-protective behaviors, and then over time, the effect wore off as participants acclimatized to wearing the device. This has been observed in the physical activity literature, for example, that the first measurement day was the most active day for participants, and this tapered off in subsequent days [20]. Future studies could further explore the role of reactivity associated with UVR monitoring devices by assessing participants over a longer wearing period and incorporating an initial familiarization phase to allow for any reactivity effects to subside. In addition, future studies could consider systematically excluding the first few days of UVR monitoring data during analysis.

We observed seasonal differences in sun-protection behaviors based on both child reports and parent reports, which is consistent with the findings of prior studies that individuals may make different sun-protection choices based on weather or time of year [14-17]. Our findings confirm the importance of controlling for seasonal effects, either through statistical methods or study design. For example, studies could take place during the course of a single season (eg, summer).

This study has several strengths and limitations. Strengths include that our study is one of the first to examine the reactivity to UVR exposure monitoring using a wearable UVR monitoring device. In addition, parents and children were included in this study, and they could both benefit from skin cancer interventions that incorporate wearable UVR monitoring devices. Limitations include a study sample from a single location, which may limit generalizability to populations in other geographic areas. Future studies could include a control group that does not wear a sensor to disentangle the potential contributions of the self-report method of assessment of sun-protection behaviors and other outcomes. Future studies could further assess potential reactivity associated with the use of wearable UVR monitoring devices that do provide feedback on exposure and account for other factors that may impact potential reactivity, such as age, amount of time spent outdoors, and geographic location.

### Conclusions

This study is among the first to assess the potential reactivity associated with UVR exposure monitoring. Reactivity effects should be further examined in both intervention and observational contexts to better understand the impact of UVR monitoring on sun-protective behaviors and other relevant clinical outcomes. Ultimately, objectively assessed UVR exposure is an important measure to be integrated into outcome assessment for future testing of skin cancer prevention interventions. In the context of intervention testing, researchers who deploy objective UVR measures may want to compare intervention outcomes between individuals who used a UVR monitor and those who did not. In addition, when examining intervention effects on objectively assessed UVR exposure, researchers may want to analyze UVR data with and without the first few days of UVR data collected to minimize potential reactivity effects.

## Abbreviations

REDCap: Research Electronic Data Capture
UVR: UV radiation
